# Making sense of the evidence in population health intervention research: building a dry stone wall

**DOI:** 10.1136/bmjgh-2020-004017

**Published:** 2020-12-09

**Authors:** David Ogilvie, Adrian Bauman, Louise Foley, Cornelia Guell, David Humphreys, Jenna Panter

**Affiliations:** 1 MRC Epidemiology Unit, University of Cambridge, Cambridge, UK; 2 School of Public Health, The University of Sydney, Sydney, New South Wales, Australia; 3 European Centre for Environment and Human Health, University of Exeter, Truro, UK; 4 Department of Social Policy and Innovation, University of Oxford, Oxford, UK

**Keywords:** prevention strategies, public health, intervention study, systematic review

## Abstract

To effectively tackle population health challenges, we must address the fundamental determinants of behaviour and health. Among other things, this will entail devoting more attention to the evaluation of upstream intervention strategies. However, merely increasing the supply of such studies is not enough. The pivotal link between research and policy or practice should be the cumulation of insight from multiple studies. If conventional evidence synthesis can be thought of as analogous to building a wall, then we can increase the supply of bricks (the number of studies), their similarity (statistical commensurability) or the strength of the mortar (the statistical methods for holding them together). However, many contemporary public health challenges seem akin to herding sheep in mountainous terrain, where ordinary walls are of limited use and a more flexible way of combining dissimilar stones (pieces of evidence) may be required. This would entail shifting towards generalising the functions of interventions, rather than their effects; towards inference to the best explanation, rather than relying on binary hypothesis-testing; and towards embracing divergent findings, to be resolved by testing theories across a cumulated body of work. In this way we might channel a spirit of pragmatic pluralism into making sense of complex sets of evidence, robust enough to support more plausible causal inference to guide action, while accepting and adapting to the reality of the public health landscape rather than wishing it were otherwise. The traditional art of dry stone walling can serve as a metaphor for the more ‘holistic sense-making’ we propose.

**Summary box**

- Systematic reviews and guidance development groups frequently conclude that the available evidence about the effects of population health interventions is too diverse, flawed or inconclusive to support a more general conclusion about what should be done.
- In spite of all the developments in quantitative methods for primary research and evidence synthesis, we struggle to derive meaningful generalisable inferences from the evaluation of interventions in arenas such as the food, transport or welfare systems to guide and support public health action.
- We respond to a long-standing call for more ‘holistic sense-making’ in this arena by proposing a more eclectic, flexible and reflexive approach to building and interpreting the evidence.
- We show how a spirit of pragmatic pluralism might be channelled into constructing ‘dry stone walls’ of evidence, robust enough to support more plausible causal inference to guide action, while accepting and adapting to the reality of the public health landscape rather than wishing it were otherwise.
- We should look beyond simple notions of ‘interventions’, search for patterns and embrace the mess in evidence synthesis in order to better understand what makes for an effective public health strategy.

## Introduction

Effectively tackling population and planetary health challenges such as climate change or diabetes requires us to address the fundamental, upstream determinants of behaviour and health in populations.^1^ This may sometimes entail contentious policies such as diverting funds from other priorities or constraining people’s freedoms, which ought to be guided by the best available scientific evidence. To this end, it is increasingly accepted that we should advocate, fund and strengthen the evaluation of interventions in arenas such as the food, transport or welfare systems, often in the form of natural experiments.^2^

However, as the ongoing drip-feed of contested and contradictory research findings in respect of coronavirus pandemic control measures has illustrated, merely increasing the supply (and rigour) of primary studies is not enough.^3^ Governments have to make decisions all the time. The pivotal link between research and policy or practice should be the cumulation of insight from multiple studies in some form of evidence synthesis,^4^ but systematic reviews and guidance development groups frequently conclude that the available evidence about the effects of population health interventions is too diverse, flawed or inconclusive to support a more general conclusion about what should be done.^2^

One reason for this is that studies conducted in ‘real-world’ settings are often critiqued for a lack of internal validity in comparison with randomised trials in more controlled settings. This may be compensated for by greater external validity—the likelihood of producing practice-based evidence that might be successfully translated to the systems in which others work.^5^ However, the fact that these studies are produced in *particular* settings is also the main apparent impediment to their generalisability. Interventions to change such things as how products are taxed, how cities are laid out or how society supports people in old age inevitably take place in particular places with particular characteristics, which vary widely across the globe and even within countries. How might we do a better job of deriving meaningful *generalisable* inferences from studies like this to guide and support public health action in other places?

## Promising solutions or false refuges

### Feeding the meta-analytical machine: piling the stack and singing in harmony

Thrombolysis was not routinely used to treat heart attack until the late 1980s. If the available trials had been combined in a meta-analysis, however, its effectiveness would have been established beyond reasonable doubt by 1973.^6^ Precedents like this suggest that the solution to a lack of evidence is simply to conduct more intervention studies in more places, on the basis that once we have a tall-enough stack of good-enough papers to populate a meta-analysis, we will know.

In practice, however, many systematic reviews of population health improvement strategies have been more successful in delineating what we do not know than in identifying unequivocally effective interventions.^2^ Often, what has prevented the formulation of clear answers is not so much a lack of studies as the lack of a way of reconciling the diversity of their study designs, limitations, interventions and contexts.^7^ One apparent solution is to limit meta-analysis to a set of more statistically comparable studies, but this risks perpetuating an evaluative bias in favour of an intervention ‘monoculture’ that may or may not include the most promising strategies.^8^ Another way of dodging the challenge is to split the problem into more and more discrete and evaluable chunks. These may eventually tell us the effect of doing X, but however refined the answers to this kind of ‘splitting’ question turn out to be, they are not sufficient to address the more pressing ‘lumping’ question for public health: how can we best achieve Y?^9 10^

If population-level intervention studies were to use a common set of exposure and outcome measures, this would make meta-analysis more feasible. Important progress has been made in this respect, for example in physical activity epidemiology.^11^ One might envisage some form of multicentre study in which more-or-less comparable interventions were introduced (or not) in different places and evaluated along the lines of a cluster randomised controlled trial. However, the Achilles heel of this vision is the qualifier ‘more-or-less comparable’. Some interventions, such as screening programmes, might be designed and implemented in a sufficiently similar way for this kind of multicentre evaluation.^12^ For upstream interventions in complex systems, however, the harmonised measurement of exposures (to interventions) and intermediate and final outcomes involving multiple causal pathways is challenging. Consider, for example, the array of measures of pricing, product formulation, purchasing, consumption, diet, health, and potentially confounding background trends that are needed to properly quantify the intended and unintended impacts of introducing a national levy on sugar-sweetened drinks.^13^ Negotiating the harmonised *implementation* of truly comparable interventions in multiple jurisdictions and beyond the control of researchers may be even less feasible.

### Broadening the scope: building the panopticon and modelling the solutions

If empirical intervention studies are so difficult to design, implement or combine in meta-analysis, why not make more use of observational and simulation methods? The growth of ‘big data’ and interest in the ‘quantified self’ now offer unprecedented possibilities to gather enormous quantities of information, whether from surveillance systems such as traffic cameras or portable devices unobtrusively capturing continuous geographical, physiological and other signals from individuals. This torrent of data has led us towards a contemporary version of Bentham’s panopticon, telling observers exactly what people are or have been doing, where, when and even with whom. Datasets of this breadth, depth and precision make it possible to investigate associations with an unprecedented degree of statistical power and analytical complexity, and one might assume that so long as sufficiently rich data are available to populate such analyses, more and more secure causal inference will follow. The results can also be used as inputs to tools such as systems dynamic modelling—to simulate the consequences of altering upstream determinants of health, identify new intervention points and explore to what extent the outcomes observed in one system may be generalisable to others.^14^

However, enthusiasm for increasing computational complexity in the search for causal inference from observational or simulation data should be tempered with the recognition that design-based inference is generally considered stronger than model-based inference.^15^ In other words, we should attend at least as much to investigating situations in which different groups are exposed to different *exogenous* factors (interventions, or at least determinants of *change*) as we do to refining ways of eliciting ‘causal’ evidence from other datasets. Even if well-founded concerns about the representativeness and privacy implications of relying on ‘big data’ can be addressed, the resulting associational cornucopia is unlikely to help much if it contributes merely to producing ‘ever more sophisticated answers to the wrong questions’.^16^

For example, hundreds of studies now tell us that more walking is reported in areas where it is easier and safer for people to walk and there are places for them to walk to.^17^ However, precisely quantifying dose–response relationships of this kind does not necessarily explain how to address the problem of comparative inactivity, just as proving the aetiological case against tobacco did not explain how to reduce the prevalence of smoking.^18^ We should therefore not assume that the answers to the question of what we should do will be found by searching for statistical associations that only become noticeable in extremely large samples. Cohort and surveillance data collected for other purposes can certainly be used to investigate the effects of interventions,^19^ but no matter how intensively people’s health, behaviour and environments are quantified in observational studies, it may be a category error to assume that this will necessarily explain whether or how public health strategies actually work (or not). Epidemiology is only one of the tools in the box.^20^

## Is the craft of evidence synthesis fit for purpose?

The point of evidence synthesis is surely to derive more generalisable causal inference. In spite of the academic language, this is as much an applied problem as an abstract, theoretical problem; to put it another way, what can transport planners in Birmingham learn from what their counterparts did in Bogotá?^5^

If cumulating evidence from multiple studies can be thought of as analogous to building a wall, then the ‘solutions’ outlined above can be regarded as ways of increasing the supply of bricks (the number of pieces of information), the similarity (statistical commensurability) of the bricks or the strength of the mortar (meta-analytical or other statistical methods for holding them together). These are helpful if the aim is to build a larger and stronger conventional wall, formed of neat rows of bricks of roughly the same shape and size.

**Figure 1:**
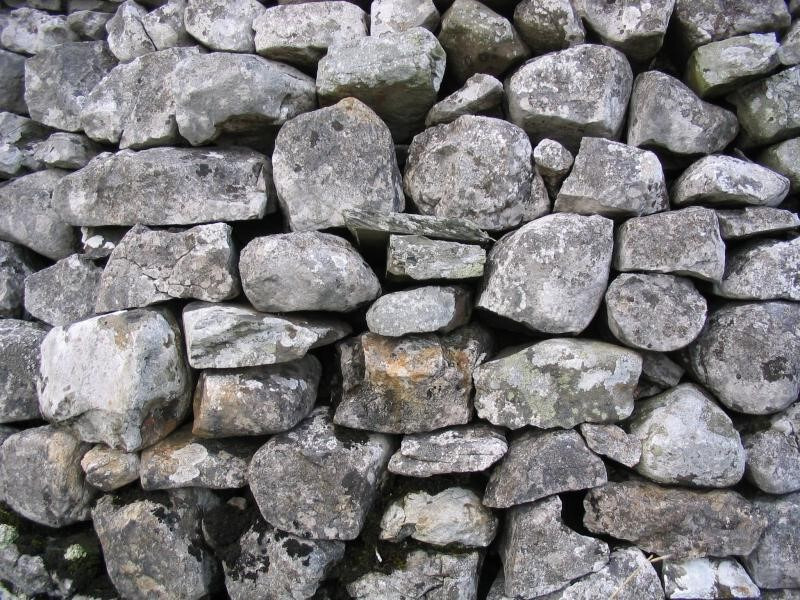
Dry stone wall. Credit: Lupin at English Wikipedia (CC BY-SA). http://upload.wikimedia.org/wikipedia/commons/1/10/Dry_stone_wall_in_the_yorkshire_dales_detail.jpg

Important and useful as all these approaches are, they have the potential to distract us from the real problem. Conventional brick walls work best on flat, smoothly prepared ground. Many contemporary public health challenges seem more akin to herding sheep in a mountainous landscape characterised by steep slopes, rocky outcrops and boggy ground. In this terrain, the more artisanal, bespoke and traditional solution of the dry stone wall may be more useful (figure 1). Dry stone walling is a way of transforming a pile of stones, which at first glance do not fit together, into something new and useful. Each stone is considered in its own right and assigned a unique place in the wall. No mortar is required, because careful thought is given to how all the pieces can be related to form a robust structure that is more than the sum of its parts. The art can be learnt, but it requires a level of flexibility and ingenuity that cannot readily be codified. It can therefore stand as a metaphor for the 'holistic sense-making' required of the evidence in population health intervention research.^21^ How might we better harness our research skills and technologies—ancient and modern—to build evidential structures more suited to the terrain we inhabit?

## Building a dry stone wall of evidence

### Looking beyond ‘interventions’

For most public health interventions—even well-established population screening programmes—the only honest answer to the question ‘Does it work?’ is ‘It depends’.^14 22^ In most cases, the questioner will need to clarify what they mean by *work* (in what terms?), what they mean by *it* (what, exactly, is the intervention?) and indeed what they mean by *does* (which implies a generalisable inference). Sometimes, what people really mean is ‘Will it work?’—a predictive question—or ‘Did it work?’—an empirical but also a *particular* question, perhaps better formulated as ‘What happened?’^10^

Why is this so difficult? Most public health interventions are at least somewhat unique to their context, which ought to be taken account of in their evaluation, and many can also be seen as interventions in complex systems.^10 12 22^ However, they do not necessarily take place *in* a context, at least not in the sense that a new clinical procedure might be introduced in a certain hospital or healthcare system. Rather than exerting an effect within a ‘moderating’ context, it may be more helpful to see these interventions as targeting and *altering* the context in which people live and make choices.^10 23 24^

But if everything is complex and context ‘is’ the intervention, what exactly might we seek to generalise from one instance to another? We tend to assume that interventions are things that work in and of themselves and might be universally generalisable, like Newton’s laws of motion.^25 26^ In practice, however, we find ourselves struggling to make sense of an apparently incommensurable body of evidence. By adopting a ‘naive and misplaced commitment to the reproducibility of the complex’, we may have unwittingly set ourselves an unrealistic challenge of identifying generalisable interventions as such.^22 27 28^ What if we were to release ‘our search for universal generalisability in favour of more modest, more contingent, claims’?^22^ Among other things, this would entail relaxing our grip on the notion of generalising the effects of interventions based on their *forms* or their ‘active components’, and turning our attention instead to their *functions*—the processes and changes they evoke—or indeed their ‘spirit’.^12 26^

To take a well-known clinical example, some reviews have found that the type of psychotherapy offered to a patient with depression makes little difference to the outcome.^29^ What to do, then? Others, taking a different approach to analysis, have found that the key lies in the quality of the therapeutic relationship established rather than the particular techniques used.^30^ Reasoning by analogy, we theorised that in another arena—promoting active travel—the myriad forms of intervention might be underlain by a more limited number of critical functions, such as increasing accessibility or safety, that are generalisable in principle but might be achieved in different ways in different situations.^31^ Again, this is as much to do with practical strategies as it is to do with theories, and some public health guidance and government policy already implicitly reflects this way of thinking with references to high-level principles, variable interpretations and the like (examples: table 1).

**Table 1:** Examples of generalisable principles reflected in policy and practice guidance

Example of guidance	Interventions considered	Main objective of guidance	Key principles of approach taken	Illustrative content of recommendations
Public health guidance from the National Institute for Health and Care Excellence^41^	‘Interventions in the built or natural environment that encourage and support physical activity among all population groups’^42^	‘How to improve the physical environment to encourage and support physical activity’	‘Even if there is a policy in place to address these issues, **the way it is interpreted and put into practice may vary** both between areas, and over time in the same area’	‘Ensure (…) modes of transport that involve physical activity are given the highest priority (…) **Use one or more of the following methods**: Reallocate road space (…) (**for example**, by widening footways and introducing cycle lanes) Restrict motor vehicle access (**for example**, by closing or narrowing roads to reduce capacity) Introduce road-user charging schemes (…) Introduce traffic-calming schemes to restrict vehicle speeds (…)
Transport network management guidance in response to COVID-19 from the Department for Transport^43^	‘Changes to (…) road layouts to give more space to cyclists and pedestrians’	‘When the country gets back to work, we need them to carry on cycling, and to be joined by millions more (and) pedestrians will need more space’	‘The guidance sets out **high-level principles** to help local authorities to manage their roads and what actions they should take’	‘Local authorities where public transport use is low should be **considering** all **possible measures** (…) None of these measures are new (…) but a step-change in their roll-out is needed to ensure a green restart. **They include:** Installing ‘pop-up’ cycle facilities (…) **for example,** mandatory cycle lanes, (…) light segregation features (or) temporary cycle lanes (…) Encouraging walking and cycling to school, **for example** through the introduction of more ‘school streets’ (…) Modal filters (also known as filtered permeability); closing roads to motor traffic, **for example** by using planters or large barriers (…)

Emphases added.

### Searching for patterns

If this idea has traction, we will need to expand the scope and flexibility of our repertoire in evidence synthesis in order to derive more concrete and defensible inferences about what to do for public health. Rather than assessing whether interventions of a particular type ‘work’ in an overall sense, this will entail aggregating evidence for and against *theories* about intervention functions by combining information from studies conducted in different situations, including studies that were not explicitly designed with this in mind.^7 14 27 32 33^

How might we do this? We could accept the limitations of relying so heavily on testing binary statistical hypotheses about singular study outcomes,^2 14^ and turn our attention to seeking ‘inference to the best explanation’—that which provides the greatest *understanding*.^18 34 35^ We could use intervention theory to predict patterns that might be observed in a variety of data, and then assess the concordance between the observed patterns and the theoretical expectation patterns—testing theories rather than interventions.^33 36^ We could go further by systematically considering alternative potential explanations for the patterns we observe, doing our best to confirm or disconfirm these, and reaching a conclusion as to the most plausible causal inference from the overall pattern of findings. The approach is most easily illustrated within a single study, an evaluation of new transport infrastructure that was not designed to test any singular overarching hypothesis (case study: table 2).

**Table 2:** Case study of the dry stone wall principle applied to an intervention study

Intervention considered	Main research question	Key principles of approach taken	Examples of key methods and findings
‘A new (…) section of (…) motorway (…) running through predominantly deprived neighbourhoods (…) with associated changes to the urban landscape’^44^	‘What are the individual, household and population impacts of a major change in the urban built environment on travel and activity patterns, road traffic accidents and well-being?’	**Looking beyond interventions**	
		‘Numerous health-related claims were made for and against the new motorway (…) We summarised these (…) as two equally valid, competing, **testable, overarching hypotheses** (…) **expressed in the form of vignettes** of two alternative extreme cases, a ‘virtuous spiral’ and a ‘vicious spiral’ (…) **using the developing situation** (…) **to understand more** about the positive and negative effects of the changes to the urban landscape’	‘Mapping our findings against the key propositions of each vignette, we find—perhaps unsurprisingly**—a mixture of confirmatory and disconfirmatory evidence** on both sides’
		**Searching for patterns**	
		‘We sought to build an evidential case for causal inference using multiple sources of data and types of analysis (…) by taking a **‘pragmatic pluralist’ approach to the ‘ragged evidence’** of the natural experimental study (…)’	‘The study used a **combination of quantitative** (cohort, cross-sectional, repeat cross-sectional and interrupted time series) **and qualitative** (documentary analysis and interview) **research methods to evaluate** both individual-level and population-level **changes** in health and health-related behaviour, **and to develop a more in-depth understanding** of how these changes were experienced and brought about’
		**Embracing the mess**	
		‘We sought to match patterns of outcomes with patterns predicted by the intervention theory imperfectly captured in these vignettes, **searching not for support for a singular overarching hypothesis, but rather for the least implausible explanation of the conditions that may be required to produce or prevent the outcomes of interest’**	‘Our evidence points to **two critical functions**—connecting and separating—that constitute two sides of the same coin and are **both evoked by the same intervention in different ways for different people** (…) The **overarching hypothesis with which our data are most consistent** is that new transport infrastructure is more likely to benefit more people when it connects people with their social and physical surroundings—broadly defined—more than it separates them, and when people are protected from its harmful environmental impact by distance or other effective mitigation measures’

Emphases added.

One might counter that the principle of comparing observed and expected data applies equally to the paradigm of the randomised controlled trial. While this is true, testing theories in the way we describe entails a more radical challenge to established notions of a hierarchy of study design. For example, it is often understood that quantitative methods are for testing hypotheses, whereas qualitative (and some quantitative) methods play more subservient roles such as generating hypotheses, developing interventions or assessing their acceptability.^32^ One might also counter that approaches such as process evaluation, or realist evaluation and synthesis, already offer ways of investigating causal mechanisms.^7 24 27 37^ While this is true, the higher-order intervention functions and data patterns we are talking about are likely to reflect multiple underlying mechanisms^25^ and others have argued that more diverse lines of evidence should be converged and brought to bear on the challenge of overall causal inference. These might combine a variety of quantitative sources of causal *estimation* with a variety of quantitative and qualitative sources of causal *explanation* such as causal process observations.^15 22 34^ Simple examples from historical and contemporary communicable disease control illustrate this principle (table 3).

**Table 3:** Examples of arguments for convergent lines of evidence in communicable disease control

Example of outbreak	Example of quantitative evidence supporting causal estimation	Example of qualitative or case-study evidence supporting causal explanation	Case for convergence
Cholera, 1854 45 46	‘Snow (…) observed that two water companies served this area (and) that households receiving water from the two companies were intermingled on the same streets. These insights provided the basis for assuming that allocation of contaminated water occurred as-if at random, thus justifying the (quantitative) **natural experiment (that) yielded an unusually decisive confirmation of his main hypothesis** (…) that cholera spread through human contact and was waterborne.’	‘Early on, he **abandoned the established explanation** that cholera spread through “miasmas”(…) Snow **observed** that (…) the first case in London involved the death of a sailor just arrived from Hamburg, where there was a cholera epidemic. The second was an individual who subsequently slept in the same boarding house room as this sailor.’	‘Snow was **‘intimately familiar** with the (…) area because of his medical practice’’, which contributed to astute **inferences** about the incidence of cholera **in particular neighbourhoods, workplaces and households** (…) Snow arrived at these insights prior to the confirmation provided by this experiment (and) the construction of this remarkable study was **heavily dependent on** (qualitative) **causal process observations.**’
COVID-19, 2020^47^	‘**Absence of trial evidence** is partly due to the fact that experimental studies of mass public health measures are usually impractical (…) There are now **many natural experiments** of the wearing of masks or face coverings in COVID-19.’	‘Another piece of evidence that covering the face could make a big difference is **super-spreader events** (…) Perhaps the most dramatic is the choir practice in Seattle, in which (…) 45 of 60 people became infected and 2 (so far) have died.’	‘I am struck by the stories they did not examine (the COVID-stricken choir, (etc)) But these stories (…) **pull together complex chains of influence** and remind us that **causality** (…) **is rarely linear** (…) All these various streams of evidence **contribute, in different ways and at different levels,** to strengthen the argument (…) As with other public health measures, we should make a decision based on an **assessment of the full body of evidence.’**

Emphases added.

### Embracing the mess

Some public health strategies will inevitably be more successful than others, and every ‘solution’ has the potential to generate more problems. Such uncertainty is—or at least should be—what drives scientific enquiry in the first place.^38^ Rather than hoping for 'a neat, coherent story’ of clear-cut outcomes from evaluation, therefore, in most cases we should *expect* confusing, divergent, mixed or unexpected patterns of results.^21^ Far from denoting that an intervention or evaluation has failed, these shed light on what really happened, whether we like it or not.^10^

While the metaphor of the dry stone wall can be applied at the level of the individual study, as shown in table 2, the mess of this apparent dissonance may be better resolved not at the level of the individual study, but by cumulating evidence from multiple studies over time within an intervention research programme or a systematic review. Our final case study illustrates how we applied the principles of linking diverse sources of evidence on causal estimation and causal explanation to identifying patterns and testing theories about intervention functions across a cumulated body of work on infrastructure to support active travel (table 4).^31^ It draws on a variety of research methods, resting on different philosophical assumptions, in pursuit of ‘clarification and insight, for which a more interpretive and discursive synthesis is needed’.^3 9^

**Table 4:** Case study of the dry stone wall principle applied to a systematic review

Intervention considered	Main research question	Key principles of approach taken	Examples of key methods and findings
‘Environmental changes aimed at encouraging walking or cycling’^31^	‘To understand how changes to the external physical environment may act to promote walking, cycling and physical activity, and why these may or may not be effective’	**Looking beyond interventions**	
		‘Rather than synthesising evidence from similar classes or forms of interventions (eg, cycle paths), it might be possible **to synthesise evidence from interventions which have the same function** (eg, interventions which change the perceived safety of cycling **regardless of the precise method used**). This **exploits the variation** in contexts where similar (but not exactly the same) interventions have been implemented.’	‘We identified **three common resources that interventions provide** to promote walking and cycling: (1) improving accessibility and connectivity; (2) improving traffic and personal safety; and (3) improving the experience of walking and cycling.’
		**Searching for patterns**	
		‘In the spirit of triangulating a range of types of evidence, we used **principles from a range of different methods** including narrative and realist reviews and qualitative analysis as recommended and used a **sequential explanatory approach** (…) We extracted information on the **evidence for effects (‘estimation’), contexts and mechanisms (‘explanation’**) and assessed credibility, and **synthesised material narratively** (…)’	‘We found some evidence that interventions were **considered with the wider physical and social system** in policy documents and qualitative or mixed-method studies. These sources of evidence are **traditionally viewed as lower quality**, and although they were few in number here, we found that they were **useful in painting a conceptually richer picture of potential contexts and mechanisms.**’
		**Embracing the mess**	
		‘We **identified common functions**—overarching themes—across these interventions (and) **synthesised** (…) combinations of contexts, mechanisms and outcomes **on a more abstract level** (…) with a focus on exploring patterns of outcomes (more successful and less successful) and on those with the strongest or most convincing evidence (…) We **distilled three potential ways** in which the interaction of an intervention’s function with different contexts may lead to processes and outcomes being enabled or disabled.’	‘The **most plausible mechanisms** concerned (1) improving accessibility and convenience of walking and cycling, and (2) reducing potential conflict between users (…) **The most effective interventions appeared to target accessibility and safety** in supportive and unsupportive individual and physical contexts.’

Emphases added.

## Conclusions

Many readers engaged with conducting, synthesising or applying the findings of population health intervention research are likely to agree with the editors of the Cochrane Handbook, who recently wrote that in spite of all the developments in quantitative methods for evidence synthesis, it is frequently still not possible for these ‘to provide insight beyond a commentary on what evidence has been identified’.^7^

We need to find a better way, otherwise merely piling up more studies may leave us confronting another kind of stack—endlessly circling the runway of a conclusion on which we never seem to have clearance to land. In this paper we have responded to a long-standing call for more ‘holistic sense-making’ in this arena. We have outlined a strategy of constructing ‘dry stone walls’ of evidence: pluralist mosaics whose strength derives from the complementarity of their components rather than being found in spite of it.^39^ This approach has the potential to be robust enough to support more plausible inference to guide action, while accepting and adapting to the reality of the public health landscape rather than wishing it were otherwise.

This will entail facing up to the challenge of working as ‘scholars, rather than just researchers’^21^—that is, artisanal dry stone wallers rather than bricklayers. We are advocating not a new method of evidence synthesis as such, but a more eclectic, flexible and reflexive approach; ‘not the abandonment of more reductive lines of research but the enlargement of these’^40^ with the more thoughtful and practical application of theory to generating practice-based evidence in public health. Ironically, it may only be by combining growing quantitative sophistication with the least technologically dependent research method of all—the anthropological tradition of the ethnographic observation of people and societies—that we will really understand what makes for an effective public health strategy.
